# Characterizing the Role of *AosfgA* and *AofluG* in Mycelial and Conidial Development in *Arthrobotrys oligospora* and Their Role in Secondary Metabolism

**DOI:** 10.3390/microorganisms12030615

**Published:** 2024-03-19

**Authors:** Qianqian Liu, Na Bai, Shipeng Duan, Yanmei Shen, Lirong Zhu, Jinkui Yang

**Affiliations:** 1 State Key Laboratory for Conservation and Utilization of Bio-Resources in Yunnan, Key Laboratory for Southwest Microbial Diversity of the Ministry of Education, School of Life Science, Yunnan University, Kunming 650032, China; liuqianqian614@163.com (Q.L.); baina_hist@163.com (N.B.); duanshipeng@stu.ynu.edu.cn (S.D.); shenyanmei@stu.ynu.edu.cn (Y.S.); zhulirong_dif5@stu.ynu.edu.cn (L.Z.); 2 School of Life Science and Technology, Henan Institute of Science and Technology, Xinxiang 453003, China

**Keywords:** *Arthrospora oligospora*, sporulation-related genes, vacuole assembly, secondary metabolism

## Abstract

*Arthrobotrys oligospora*, a widespread nematode-trapping fungus which can produce conidia for asexual reproduction and form trapping devices (traps) to catch nematodes. However, little is known about the sporulation mechanism of *A. oligospora*. This research characterized the functions and regulatory roles of the upstream spore-producing regulatory genes, *AosfgA* and *AofluG*, in *A. oligospora*. Our analysis showed that *AosfgA* and *AofluG* interacted with each other. Meanwhile, the *AofluG* gene was downregulated in the Δ*AosfgA* mutant strain, indicating that *AosfgA* positively regulates *AofluG*. Loss of the *AosfgA* and *AofluG* genes led to shorter hyphae and more septa, and the Δ*AosfgA* strain responded to heat and chemical stresses. Surprisingly, the number of nuclei was increased in the mycelia but reduced in the conidia of the Δ*AosfgA* and Δ*AofluG* mutants. In addition, after nematode induction, the number and volume of vacuoles were remarkably increased in the Δ*AosfgA* and Δ*AofluG* mutant strains. The abundance of metabolites was markedly decreased in the Δ*AosfgA* and Δ*AofluG* mutant strains. Collectively, the *AosfgA* and *AofluG* genes play critical roles in mycelial development, and they are also involved in vacuole assembly, the stress response, and secondary metabolism. Our study provides distinct insights into the regulatory mechanism of sporulation in nematode-trapping fungi.

## 1. Introduction

Plant-parasitic nematodes are widely distributed and are the pathogens responsible for numerous crop diseases and yield reductions, which seriously impair agricultural production [1]. At present, the disease control of plant-parasitic nematodes is still dominated by chemicals. However, chemicals are not only highly toxic, but also potentially harmful to both organisms and the environment [2]. In recent years, research on efficient and environmentally friendly nematicidal bioresources has received increasing attention. Nematode-trapping fungi (NTFs) are a class of filamentous fungi that produce specialized traps to capture and digest nematodes [3]. Currently, diverse traps have been discovered in various NTFs, including adhesive three-dimensional networks, adhesive branches, adhesive knobs, constrictor rings, non-constricting rings [4,5], acanthocytes [6], and spiny balls [7]. Nematodes can be captured through adhesion or mechanical means. The nematode’s mycelium forms specialized invasive structures when the fungus contacts the nematode and subsequently destroys the nematode cuticle through mechanical expansion accompanied by the secretion of degrading enzymes [8]. Moreover, endoparasitic fungi attack nematodes through spores, which germinate rapidly, and after being ingested by the nematode, the mycelium invades the internal structure of the nematode, leading to nematode death [9].

*Arthrobotrys oligospora* is a classic NTF that mainly produces adhesive three-dimensional networks to adhere, capture, penetrate, infest, and disintegrate nematodes after sensing their presence [10]. To efficiently capture “prey”, *A. oligospora* has evolved to attract nematodes using olfactory mimicry and sex pheromones [11]. *A. oligospora* expands its reproduction mainly by producing conidia. Interestingly, the conidia of *A. oligospora* display significant variations in size and morphology on different media [12]. In addition, under specific survival conditions, such as feces and soil where plants live, the conidia of *A. oligospora* can germinate directly to form conidial traps without moving through the nutrient growth stage [13,14]. Therefore, the study of the regulatory mechanism of sporulation is crucial for elucidating the mycelial development, trap formation, and potential application of *A. oligospora* and other NTFs.

Spore production involves multiple biological processes, such as gene expression, cell differentiation, and cellular interrelationships [15]. Notably, asexual spore production is essential to the lifecycle of most filamentous fungi [16]. Presently, there are few studies on the functions and regulatory mechanisms of the spore-producing regulatory genes of NTF. However, the regulatory mechanisms of sporulation have been elucidated in the model fungi *Aspergillus nidulans* and *Neurospora crassa*. In *A. nidulans*, the *brlA*, *abaA*, and *wetA* genes together form a central developmental pathway (CDP), which mainly regulates the orderly expression of other sporulation-related genes [17]. All three genes are essential for spore production [18,19]. In addition, an upstream developmental activation pathway (UDAP), including *fluG*, *flbA*, *flbB*, *flbC*, *flbD*, and *flbE*, can lead to the initiation of sporulation and the activation of *brlA* [20,21]. *SfgA*, a negative regulator, represses *fluG* and *flbA*-*flbE* during the trophic growth phase. Later, *fluG* gradually releases the inhibitory effect of *sfgA* and activates the *flb* gene to initiate spore production [22]. Further, the velvet regulator *vosA* and *velB* genes were able to be activated by *abaA*, whereas *brlA* was regulated via negative feedback from the *velB*-*vosA* heterodimer [23]. Notably, light-dependent regulatory networks (*fphA*, *lreA*, *lreB*, *flbA*, *flbB*, and *flbC*) and G-protein regulatory networks (*fadA*, *sfaD*, and *gpgA*) were also reported in *A. nidulans* [24,25]. The two pathways differ in that light-dependent regulatory networks primarily regulate the ratio of asexual and sexual development in fungi, whereas G-protein regulatory networks determine between trophic mycelial growth and asexual spore production development [26,27]. Unlike *A. nidulans*, the genes which participate in the regulation of macroconidia formation are *acon-2*, *con-3*, *csp-1*, and *csp-2* in *N. crassa* [23]. In summary, asexual spore production in filamentous fungi is a complex process co-regulated by multiple genes. The study of the asexual spore production mechanism of NTFs and other biocontrol fungi can lay the foundation for the study of mycelial growth, development, and differentiation mechanisms, and also provide new insights into the development of efficient nematode bio-control agents.

In recent years, studies on *A. oligospora* have centered on mycelial development and trap formation. However, limited studies have been conducted on spore-producing genes. Previous studies show a close link between traps and conidia, and the knockout of most signaling proteins or functional genes affects not only the trap formation but also the formation of conidia [28,29,30]. Recently, our group has elucidated the functions of AoMedA, AoBrlA, AoAbaA, and AoWetA in *A. oligospora*. *AomedA*, *AobrlA*, *AoabaA*, and *AowetA* serve crucial roles in the spore formation, mycelial development, trap formation, and vacuole assembly of *A. oligospora* [31]. However, the functions of the UDAP-associated genes are still unknown in NTF. In the current research, we investigated the interactions and roles of the UDAP genes *AosfgA* and *AofluG* in *A. oligospora*. Our data suggest that *AosfgA* and *AofluG* are involved in mycelial growth, conidium formation, the stress response, vacuole assembly, and secondary metabolism in *A. oligospora*.

## 2. Materials and Methods

### 2.1. Strains, Plasmids, and Growth Conditions

*Arthrobotrys oligospora* wildtype (WT) strain (ATCC 24927) and the mutant strains Δ*AosfgA* and Δ*AofluG* were cultured on potato dextrose agar (PDA) (200 g potato, 20 g dextrose, and 20 g agar per 1 L) medium at 28 °C. *Saccharomyces cerevisiae* strain FY834, a uracil-deficient strain, was used for knockout vector construction and screening and was cultured on yeast extract peptone dextrose (YPD) (10 g yeast extract, 20 g peptone, and 20 g dextrose, and 20 g agar per 1 L) medium at 30 °C. In addition, the *Escherichia coli* strain (DH5α) (TaKaRa Biotechnology Co. Ltd., Dalian, China) was utilized for the preservation and cloning of plasmid vectors. It was grown on Luria–Bertani (LB) medium at 37 °C [32]. Further, pCSN44 and pRS426 plasmids were used to amplify the hygromycin cassette (*hph*) and construct the knockout vectors, respectively [33]. *Caenorhabditis elegans* N2 was cultivated in an oat medium at 26 °C for the induction of trap formation of the NT fungi.

### 2.2. Fluorescent Quantitative PCR (RT-qPCR)

Mycelial samples of the WT strain cultured from 1 to 7 days on corn dextrose with yeast extract (CMY) (20 g corn starch, 5 g yeast extract, and 20 g agar per 1 L) medium were collected, and mycelial RNA was extracted using the Multisource Total RNA Miniprep Kit (Axygen Scientific, Union City, CA, USA). Subsequently, cDNA was obtained using PrimeScript RT Reagent Kit with gDNA Eraser (TaKaRa Biotechnology Co. Ltd., Dalian, China). Finally, RT-qPCR was performed according to the manufacturer’s instructions. The 2^−ΔΔCt^ method was used to analyze the obtained data, and the β-tubulin gene (AOL_s00076g640) was used as the reference [34]. The primers for detecting transcript levels of sporulation-related genes are shown in Table S1. The relative expression level (RTL) levels of the genes were presented using GraphPad Prism 8.0 (GraphPad Software Inc., San Diego, CA, USA). Three biological replications were performed for the experimental data.

### 2.3. Y2H Assay

The *AofluG*, *AosfgA*, *AoflbA*, and *AovosA* genes were ligated to pGADT7 and pGBKT7 vectors, respectively, and then the above vectors were cotransformed into Y2HGold yeast competent cells (Clontech, CA, USA). Meanwhile, pGBKT7-53/pGADT7-T and pGBKT7-Lam/pGADT7-T vectors were used as positive and negative controls, respectively. The specific experimental methods and medium selection were the same as described previously [35].

### 2.4. Sequence and Phylogenetic Analysis of AoSfgA and AoFluG

Using the amino acid sequences of spore-producing genes in model fungi such as the *Aspergillus nidulans*, *Aspergillus fumigatus*, and *Neurospora crassa* as references, the homologs of *AosfgA* and *AofluG* in *A. oligospora* were obtained through comparison using the NCBI database [36]. The physicochemical properties of individual amino acid sequences were calculated using ExPASy-ProtParam-tool, and the domains and functional sites of proteins were predicted using InterProScan. After that, the amino acid sequences were compared and analyzed by using DANMAN (version 6.0) (Lynnon Biosoft, San Ramon, CA, USA) and Clustal X (version 1.81) software in combination. The neighbor-joining (NJ) trees were constructed using MEGA 7.0 and subjected to 1000 bootstrap replicates [37].

### 2.5. Targeted Gene Deletion

The sequences of *AosfgA* (AOL_s00097g406) and *AofluG* (AOL_s00043g361) were obtained from the genome of *A. oligospora* via the NCBI database. The upstream and downstream fragments of *AosfgA* and *AofluG* were PCR-amplified from *A. oligospora* using paired primers, and the *hph* gene was amplified using the pSCN44 plasmid as a template. The primers for gene disruption are listed in Table S2. The PCR amplicons and pRS426 plasmid backbone (digested with *Eco*RI and *Xho*I) were co-transformed into *S. cerevisiae* FY834 by electroporation and the recombinant clone strains pRS426-*AosfgA*-*hph* and pRS426-*AofluG*-*hph* were screened on SC-Ura medium. Afterward, the fragments were transformed into protoplasts of *A. oligospora* by a PEG/CaCl2-mediated method [31]. The positive transformants were screened using PDAS (200 g potato, 0.3 g yeast extract, 0.6 M sucrose, 10 g molasses, and 20 g agar per 1 L) containing 200 mg/mL hygromycin (Amresco, Solon, OH, USA) and verified by means of PCR and Southern blotting (digested with *Hind*III) [38]. Southern blotting was performed using the North2South Chemiluminescent Hybridization and Detection Kit (Pierce, Rockford, IL, USA) according to the manufacturer’s instructions.

### 2.6. Colony Growth and Stress Adaption Analysis

To assess mycelial growth, WT, Δ*AosfgA*, and Δ*AofluG* strains were inoculated on PDA and TG media at 28 °C for 5 days, and then the mycelial growth rate was observed and recorded. The heat tolerance of the strains was tested by using the following method: WT and mutant strains were incubated on TYGA (10 g tryptone, 5 g yeast extract, 10 g dextrose, 5 g molasses, and 20 g agar per 1 L) medium at 28 °C for 1 d; then, they were placed in the incubator at 28 °C, 34 °C, 38 °C, 40 °C, 42 °C, and 44 °C for 8 h, respectively. Finally, the plates were incubated at 28 °C until day 5, after which the diameters of the colonies were measured [30]. The sensitivities of the WT, Δ*AosfgA*, and Δ*AofluG* strains to different stresses were as follows: H_2_O_2_ (5, 10, and 15 mM) and menadione (0.05, 0.07, and 0.09 mM) as oxidative stressors, NaCl (0.1, 0.2, and 0.3 M) and Sorbitol (0.25, 0.5, and 0.75 M) as osmotic stressors, and SDS (0.01%, 0.02%, and 0.03%) and Congo red (0.03, 0.06, and 0.09 mg/mL) as cell wall-perturbing agents [39]. Briefly, the WT and mutant strains were inoculated in TG (10 g tryptone, 10 g dextrose, and 20 g agar per 1 L) medium and treated with various chemical stress reagents for 5 days at 28 °C. The type and concentration of the stressor are marked in the images. After that, the colony diameter was determined, and the relative growth inhibition (RGI) was determined [40].

### 2.7. Staining Analysis

To examine the changes in mycelial cells and conidia, septa and nuclei were stained using 20 μg/mL calcofluor white (CFW) (Sigma-Aldrich, St. Louis, MO, USA) and 20 μg/mL 4′, 6-diamidino-2-phenylindole (DAPI) (Sigma-Aldrich, St. Louis, MO, USA) in the dark for 5–10 min, respectively. The stained samples were observed by means of fluorescence microscopy (ECLIPSE Ni-E, Nikon, Tokyo, Japan) [28].

### 2.8. Analysis of Conidial Production and Morphology

To observe the conidiophores, the WT and mutant strains were inoculated separately on PDA medium for 3 days, then transferred to water agar (WA) plates for incubation for 24 h to 48 h, and then observed under a light microscope (Olympus, Tokyo, Japan) [41].

To determine the conidial production, the WT and mutant strains were cultured on CMY media for 7 days, and the spores were eluted with 5 mL ddH_2_O. After that, the 1 μL of the spore suspension was observed and counted using a light microscope [42]. The spore production was calculated three times.

### 2.9. Observation of Trap Morphology and Determination of Pathogenicity

A total of 2 × 10^4^ spores of the WT and mutant strains were inoculated separately on WA plates at 28 °C for 4–5 days, and approximately 200 nematodes (*C. elegans* N2) were added for trap induction. Afterward, the traps were photographed and counted at 12, 24, 36, and 48 h. Meanwhile, the mortality rates of the nematodes at different time points were characterized [43]. Three biological replicates were performed in the above experiment.

To further observe the internal structural changes in the traps of WT and mutant strains, the mycelium was collected and fixed using an electron microscope fixative (Servicebio, Wuhan, China), after which it was observed by means of transmission electron microscopy (TEM) (JEM-1400Plus, Hitachi, Japan) [44].

### 2.10. Liquid Chromatography–Mass Spectrometry (LC-MS) Analysis

The WT and mutant strains were shaken in potato dextrose broth (PDB) medium at 28 °C and 180 rpm for 5 days, and the fermentation broth was collected by filtering the mycelia. After that, the mycelia were dried and weighed [30]. An equal volume of ethyl acetate was added to the fermentation broth, sonicated three times, and left overnight. The upper organic phase was dried in a vacuum rotary evaporator, and the sample was dissolved in chromatographic-grade methanol. Then, the solution was filtered through a 0.22 μm membrane filter. Finally, the previously mentioned procedures for sample quantification, processing (Thermo Fisher Scientific Dionex Ultimate 3000 UHPLC system and a Thermo Fisher high-resolution Q precision focusing mass spectrometer) (Thermo Fisher Scientific, Miami, FL, USA), and metabolomic data (Compound Discoverer 3.3 software package) analysis were performed [45].

### 2.11. Statistical Analysis

Each experiment was biologically replicated three or more times to verify the accuracy of the results, and the data are expressed as mean ± standard deviation (SD). Prism 8.0 (GraphPad Software, San Diego, CA, USA) was used as the data analysis software. *p* < 0.05 was used as the threshold for determining significant differences, and *p* < 0.001 represented highly significant differences.

## 3. Results

### 3.1. AoSfgA Interacts with AoFluG

**Figure 1:**
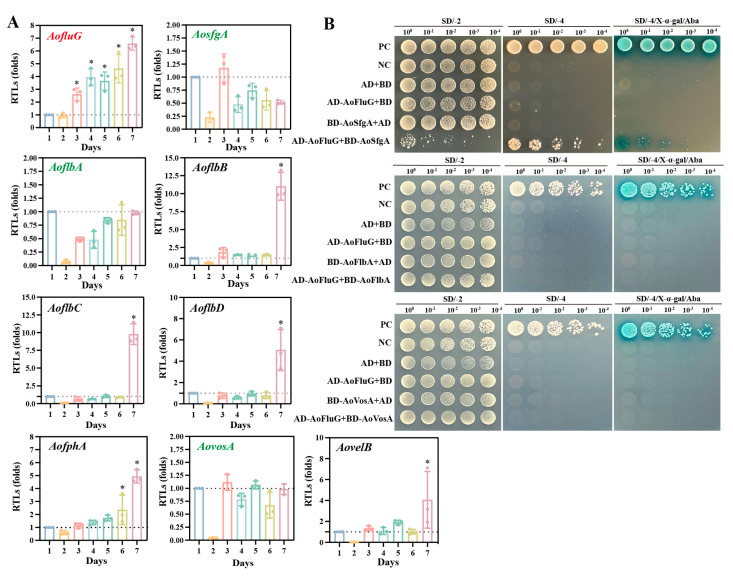
Identification of *AosfgA* interaction with *AofluG*. (**A**) RT-qPCR detection of the change in the expression level of sporulation-related genes in *A. oligospora*. The CK with the relative expression level of 1 was used as the control. (**B**) Yeast two-hybrid (Y2H) assay of *AoFluG*, *AoSfgA*, *AoFlbA*, and *AoVosA* proteins in *A. oligospora*. Plasmids pGBKT7-53 and pGADT7-T served as positive controls (PCs), whereas pGBKT7-Lam, pGADT7-T, pGBKT7, and pGADT7 served as negative controls (NCs). Yeast transformants were diluted in 0.9% NaCl, and on this basis, they were diluted four times with equal volume for 10^0^, 10^−1^, 10^−2^, 10^−3^, and 10^−4^. Growth was determined on SD/–Trp/–Leu (SD/−2), SD/–Trp/–Leu/–His/–Ade (SD/−4), and SD/−4/X−a−Gal/Aba media with serially diluted yeast cells. Asterisks indicate that the mutant strain significantly differs from the WT strain (Tukey’s HSD, * *p* < 0.05).

To ascertain the expression of the upstream regulatory genes (*AofluG*, *AosfgA*, *AoflbA*, *AoflbB*, *AoflbC*, and *AoflbD*), the light-regulated gene (*AofphA*), and the velvet regulator genes (*AovosA* and *AovelB*) during spore production in *A. oligospora*, we examined the transcriptional levels of these genes in WT strains 1–7 days post-incubation (dpi) using RT-qPCR. The findings indicated that the expression level of *AofluG* was dramatically raised from the third to the seventh dpi, whereas the expression levels of *AosfgA*, *AoflbA*, and *AovosA* were decreased or unimpaired during the culture period. In addition, the expression levels of *AoflbB*, *AoflbC*, *AoflbD*, *AofphA*, and *AovelB* initially showed a decreasing trend but increased significantly at seven dpi; in particular, the *AoflbB*, *AoflbC*, and *AoflbD* showed a similar transcription profile (Figure 1A). Next, we verified whether there was a link between the proteins with opposite expression levels using Y2H analysis. We found that *AoFluG* interacts with *AoSfgA* but not with *AoFlbA* or *AoVosA* (Figure 1B).

### 3.2. Sequence Analysis of AoSfgA and AoFluG Protein

**Figure 2:**
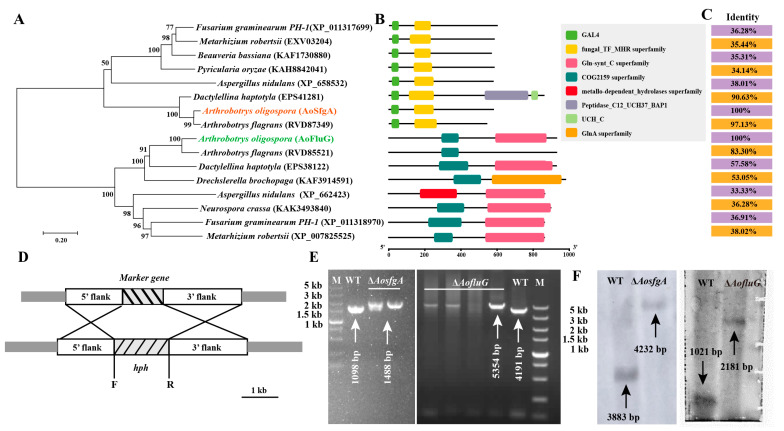
Phylogenetic analysis of *AoSfgA* and *AoFluG* proteins from different fungi and target gene deletion and validation in *A. oligospora*. (**A**) Neighbor-joining phylogenetic tree of *AosfgA* and *AofluG* homologs from different fungi. Numbers below nodes indicate the bootstrap value. The bar marker indicates the genetic distance, proportional to the number. (**B**) The conservative domains of *AosfgA* and *AofluG* homologs in different fungi. The structural domains of these sequences were analyzed using the NCBI-Batch CD-Search website. (**C**) The sequence similarity of *AosfgA* and *AofluG* homologous proteins from different fungi. (**D**) Diagrammatic sketch of homologous recombination. (**E**) PCR validation of Δ*AosfgA* and Δ*AofluG* transformants. (**F**) Southern blot analysis of Δ*AosfgA* and Δ*AofluG* transformants. WT indicates the wildtype strain.

To explore the interaction between *AoSfgA* and *AoFluG* proteins, the homologs of the two proteins were analyzed in *A. nidulans* and *N. crassa*, as well as in other NTFs. As the evolutionary tree illustrates, the homologs of *AoSfgA* and *AoFluG* are divided into two located branches, whereas both of them together were clustered with other NTFs (*Arthrobotrys flagrans*, *Dactylellina haptotyla*, and *Drechslerella brochopaga*) in the same branch (Figure 2A). The structural domains of these two proteins are highly conserved in different fungi. Among them, the major conserved domains are the GAL4 and fungal_TF_MHR superfamily in the *AoSfgA* and homologous proteins, while the major conserved domains are the COG2159 superfamily and GIn-synt C superfamily in the *AoFluG* and homologous proteins (Figure 2B). Additionally, there are higher sequence similarities of *AoSfgA* and *AoFluG* proteins with the homologs from NTFs than with other filamentous fungi; for example, *AoSfgA* shares similarities with NTFs (90.63–97.13%) and other filamentous fungi (34.14–38.01%). Similarly, *AoFluG* shares similarities with NTFs (53.05–83.30%) and other filamentous fungi (33.33–38.02%) (Figure 2C).

### 3.3. Deletion and Validation of the AosfgA and AofluG Genes

To further investigate the genes’ functions, *AosfgA* and *AofluG* genes were knocked out by means of homologous recombination (Figure 2D). The resulting transformants were confirmed via PCR to be 1098 bp and 1488 bp in size for the WT and Δ*AosfgA* mutant strains and 4191 bp and 5354 bp for the WT and Δ*AofluG* mutant strains, respectively (Figure 2E). Next, the positive transformants were further verified using Southern blotting (digested with *Hin*d III), which showed that the Δ*AosfgA* and Δ*AofluG* mutant strains had been successfully obtained (Figure 2F).

### 3.4. AosfgA and AofluG Genes Regulate Mycelial and Nuclei Development

**Figure 3:**
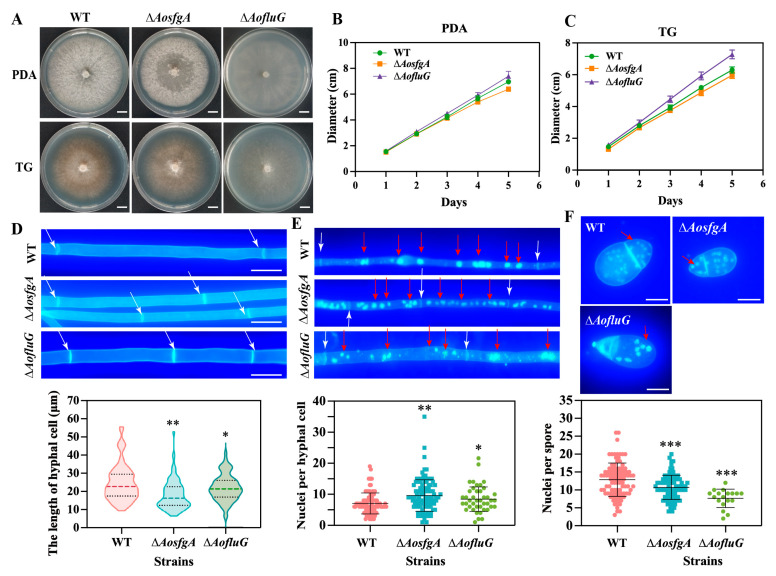
Characterization of the mycelial growth and nuclei of WT, Δ*AosfgA*, and Δ*AofluG* mutant strains. (**A**) Comparison of colony morphology on PDA and TG media. Bar = 1 cm. (**B**,**C**) Comparison of the mycelial growth rate in PDA (**B**) and TG (**C**) media for 5 days. (**D**) CFW staining observations and length statistics of mycelial cells of WT, Δ*AosfgA*, and Δ*AofluG* mutant strains. White arrow: mycelial septa. (**E**,**F**) Nuclei of mycelia (**E**) and conidia (**F**) were observed and counted by co-staining them with DAPI and CFW. White arrow: mycelial septa. Red arrow: nuclei. Asterisks indicate that the mutant strain significantly differs from the WT strain (Tukey’s HSD, * *p* < 0.05, ** *p* < 0.01, *** *p* < 0.001).

The WT and mutant strains were grown on PDA and TG plates for 6 days, and the results showed that the Δ*AosfgA* mutant strain had lower growth rates than the WT strain, whereas the Δ*AofluG* mutant strain had a higher growth rate than the WT strain, but none of them were statistically different (Figure 3A–C). CFW staining revealed that the mycelial cell septa were increased, and the average mycelial length was shorter in the Δ*AosfgA* and Δ*AofluG* mutant strains (Figure 3D). Furthermore, DAPI staining revealed many more nuclei in the mycelium of the Δ*AosfgA* and Δ*AofluG* mutant strains relative to the WT strain (Figure 3E), whereas the average number of nuclei within the conidia of the mutant strains was reduced (Figure 3F). Overall, the *AosfgA* and *AofluG* genes affect the mycelial development and the number of nuclei in *A. oligospora*.

### 3.5. AosfgA and AofluG Genes Impair Conidial Growth

**Figure 4:**
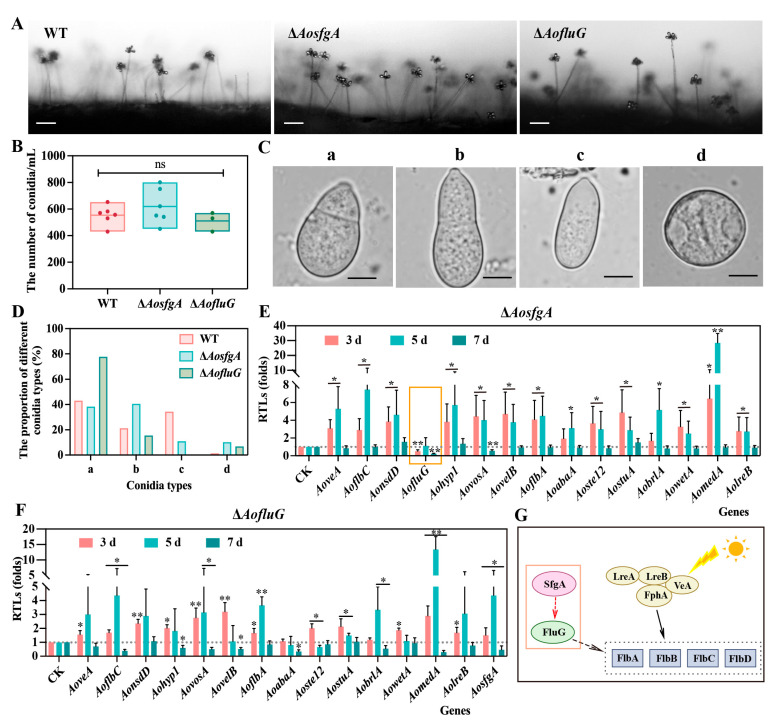
The role of *AosfgA* and *AofluG* genes on conidial development in *A. oligospora*. (**A**) Comparison of conidiophores in WT, Δ*AosfgA*, and Δ*AofluG* mutant strains. Bar = 50 μm. (**B**) Comparison of conidia yields in WT, Δ*AosfgA*, and Δ*AofluG* mutant strains. (**C**,**D**) Observations on (**C**) and statistics (**D**) for conidial styles of WT, Δ*AosfgA*, and Δ*AofluG* mutant strains. a–d in (**C**) represent different morphologies of conidia, respectively. (**E**,**F**) The expression levels of sporulation-related genes in Δ*AosfgA* (**E**) and Δ*AofluG* (**F**) mutant strains were detected using RT-qPCR, respectively. (**G**) Speculation on the interaction pattern of the upstream spore-production regulatory network of *A. oligospora*. Asterisks indicate that the mutant strain significantly differs from the WT strain (Tukey’s HSD, * *p* < 0.05, ** *p* < 0.01).

The WT, Δ*AosfgA*, and Δ*AofluG* mutant strains were grown on PDA plates for 3 days and then transferred to WA plates for conidial observation. Under the same culture conditions, the morphologies of the conidiophores of the Δ*AosfgA* and Δ*AofluG* mutants were unchanged relative to the WT strain; however, the growth densities of the conidiophores were increased (Figure 4A), whereas the conidium yields of the Δ*AosfgA* and Δ*AofluG* mutants were not statistically different from that of the WT strain (Figure 4B). The morphologies of the conidia were observed and counted, and the conidia of the WT strain were mainly of the types a, b, and c, with the most mature septate being type a (43%) and the least immature, type d, being only 1.3%. More conidia with septa were found in the Δ*AosfgA* and Δ*AofluG* mutant strains of types a (38.3% and 77.8%) and b (40.5% and 15.5%), and there were more immature conidia of type d (10.2% and 6.8%) than in the WT strain (Figure 4C,D). The above results suggest that deletion of the *AosfgA* and *AofluG* genes delays conidial formation.

### 3.6. AosfgA Positively Regulates AofluG in A. oligospora

Next, the expression levels of 15 spore-producing genes in the Δ*AosfgA* and Δ*AofluG* mutant strains were detected by means of RT-qPCR. Notably, the transcript levels of all spore-producing genes were elevated in the Δ*AosfgA* mutant strain at the third and fifth days, but the expression level of *AofluG* was remarkably reduced at the third day and had no difference from the WT strain at the fifth day (Figure 4E). In contrast, the expression levels of almost all genes were increased at the third and fifth days, except the gene *AoabaA*, which was downregulated at the seventh day in the Δ*AofluG* mutant strain (Figure 4F). Together with the previous reports, we speculate that *AofluG* is located downstream of *AosfgA* in *A. oligospora* and upstream of the *AoflbA*, *AoflbB*, *AoflbC*, and *AoflbD* genes, which further regulate sporulation (Figure 4G). Thus, it is further validated that *AosfgA* positively regulates *AofluG* in *A. oligospora* and that *AosfgA* is a critical regulator upstream of *AofluG*.

### 3.7. AosfgA and AofluG Genes Do Not Affect Trap Formation but Regulate Vacuole Assembly

**Figure 5:**
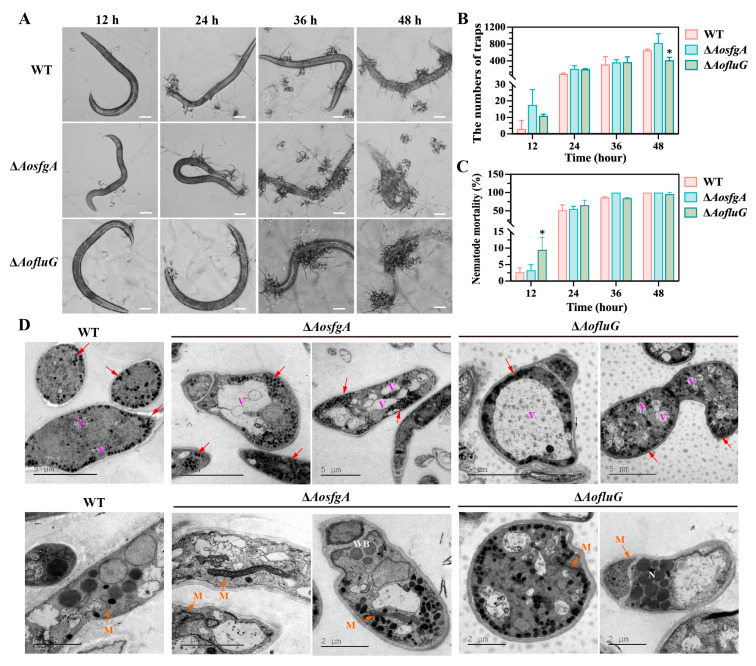
Detection of trap formation and observation of trap ultrastructure in WT, Δ*AosfgA*, and Δ*AofluG* mutant strains. (**A**) Trap formation and nematode predation by WT, Δ*AosfgA*, and Δ*AofluG* mutant strains at 12, 24, 36, and 48 h. Bar = 50 μm. (**B**) Statistics of the number of traps at different time points. (**C**) Statistics of the nematode mortality at different time points. (**D**) TEM observation of the ultrastructure of trap cells. Red arrows, electronic dense body (EDs); V, vacuole; M, mitochondrion; WB, woronin body; N, nucleus; bar = 5 μm and 2 μm. Asterisks indicate that the mutant strain significantly differs from the WT strain (Tukey’s HSD, * *p* < 0.05).

To verify whether *AosfgA* and *AofluG* affect trap formation, traps were induced by adding the same number of nematodes to the WT, Δ*AosfgA*, and Δ*AofluG* mutant strains (Figure 5A). According to the data, both the number of traps and nematode mortality in the Δ*AosfgA* and Δ*AofluG* mutant strains were a little higher than those in the WT strain, although not statistically different (Figure 5B,C). Moreover, a notable rise in the quantity and volume of vacuoles in the Δ*AosfgA* and Δ*AofluG* mutant strains was observed by means of TEM after nematode induction, in contrast to the WT strain. It was also observed that mitochondria became elongated in the Δ*AosfgA* and Δ*AofluG* mutant strains (Figure 5D). The above results demonstrate that deletion of the *AosfgA* and *AofluG* genes does not affect trap formation but affects the vacuole assembly and mitochondrial morphological changes in trap cells.

### 3.8. AosfgA Responds to Heat and Chemical Stresses

**Figure 6:**
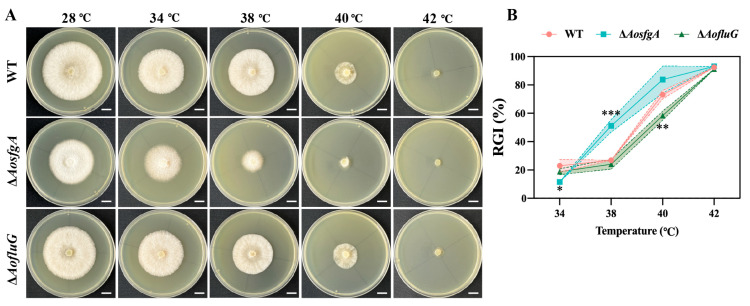
Comparison of the temperature tolerance of the WT, Δ*AosfgA*, and Δ*AofluG* mutant strains. (**A**) Comparison of the colonial morphology under high-temperature stress between the WT, Δ*AosfgA*, and Δ*AofluG* mutant strains. (**B**) RGI values of the WT, Δ*AosfgA*, and Δ*AofluG* mutant strains grown at 28, 34, 38, 40, 42, and 44 °C, respectively. Asterisks indicate that the mutant strain significantly differs from the WT strain (Tukey’s HSD, * *p* < 0.05, ** *p* < 0.01, *** *p* < 0.001).

Firstly, the WT, Δ*AosfgA*, and Δ*AofluG* mutant strains were tested for high-temperature tolerance. As can be seen from the results, the Δ*AosfgA* strain was more sensitive to high temperature, and the RGI increased significantly at 38 °C and 40 °C. In contrast, the Δ*AofluG* strain reflected insensitivity to high temperature (Figure 6A,B). Next, chemical stresses were determined for the WT, Δ*AosfgA*, and Δ*AofluG* mutant strains. Adding different concentrations of oxidative stress reagents showed that the Δ*AosfgA* strain was more sensitive to 5 mM H_2_O_2_, with an RGI value that was 1.27 times greater than that of the WT strain (Figure S1A,B). Nevertheless, the Δ*AofluG* strain was insensitive to H_2_O_2_ and menadione (Figure S1A–C). The Δ*AosfgA* strain was more sensitive to high amounts of NaCl (0.2 and 0.3 M), with 1.90- and 1.21-times higher RGI values than those of the WT strain, respectively (Figure S2A,B). In addition, the Δ*AofluG* strain was more sensitive to 0.5 M sorbitol (Figure S2A–C). Finally, in the existence of cell wall synthesis disruptors, both the Δ*AosfgA* and Δ*AofluG* mutant strains showed insensitivity to SDS, with the Δ*AosfgA* strain being sensitive to different concentrations of congo red (Figure S3A–C). Overall, the Δ*AosfgA* strain was more responsive to heat and chemical stresses than the Δ*AofluG* strain.

### 3.9. AosfgA and AofluG Genes Contribute to Secondary Metabolite Synthesis

**Figure 7:**
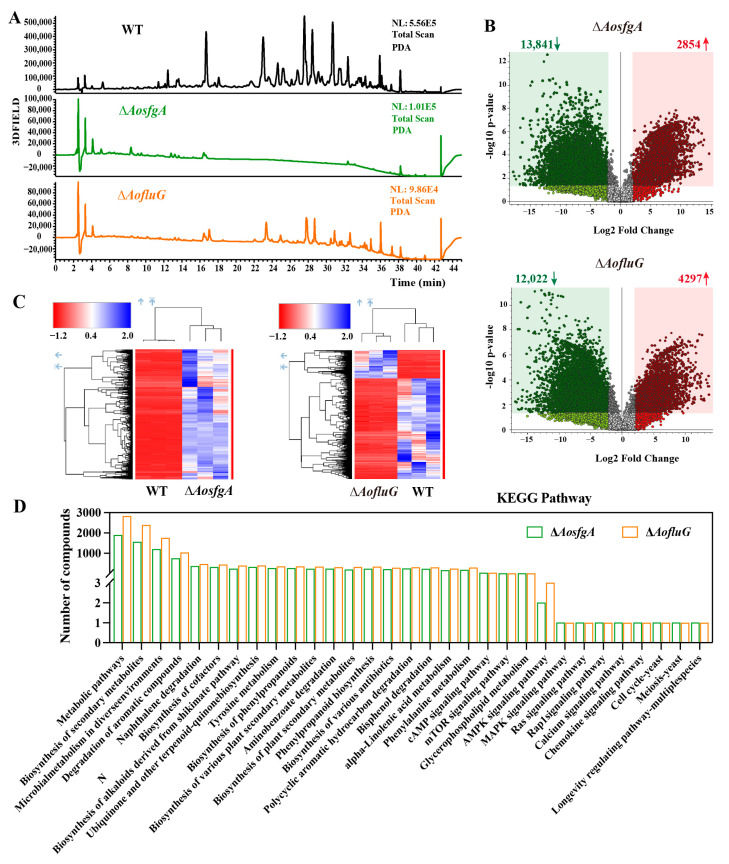
Comparison of metabolic profiling between the Δ*AosfgA* and Δ*AofluG* mutant strains. (**A**) Chromatogram analysis of secondary metabolites of WT, Δ*AosfgA*, and Δ*AofluG* mutant strains. (**B**) Volcanic map analysis of different secondary metabolites between the WT, Δ*AosfgA*, and Δ*AofluG* mutant strains. (**C**) Heat map analysis of upregulated compounds in the Δ*AosfgA* and Δ*AofluG* mutant strains, compared with the WT strains. (**D**) KEGG pathway analysis of differential compounds between the WT, Δ*AosfgA*, and Δ*AofluG* mutant strains.

The fermentation broths of the WT, Δ*AosfgA*, and Δ*AofluG* mutant strains were subjected to LC-MS. The PDA chromatograms showed a considerable reduction in metabolite abundance in the mutant strains under the same circumstances as the WT strains, with the most pronounced reduction being in the Δ*AosfgA* mutant strain (Figure 7A). The differential compounds were counted. The number of compounds upregulated and downregulated in the Δ*AosfgA* mutant strain was found to be 2854 and 13,841, respectively, while in the Δ*AofluG* mutant strain, it was 4297 and 12,022, respectively (Figure 7B). Cluster analysis of the upregulated compounds showed high expression in the WT and Δ*AofluG* mutant strains (Figure 7C). The top 20 compounds with significant changes in the Δ*AosfgA* and Δ*AofluG* mutant strains are listed in Table S3 and Table S4, respectively. Among them, the major compounds in the Δ*AosfgA* mutant strain were prednisone, diamino-N-carbamoylmethaniminium, 2-hydroxy palmitic acid, etc., while the major compounds in the Δ*AofluG* mutant strain were 3′-angeloyloxy-2′,4′-dihydroxy-6′-methoxychalcone, trichurusin F, 1H-imidazole-4,5-dicarbohydrazide, and so on. Then, KEGG pathway analysis was performed on the differential compounds. The findings demonstrated that the differential compounds were primarily concentrated in various metabolic pathways, such as secondary metabolite biosynthesis, microbial metabolism in diverse environments, tyrosine metabolism, and the degradation of aromatic compounds. Meanwhile, a few compounds were also enriched in the AMPK signaling pathway, MAPK signaling pathway, calcium signaling pathway, and cell cycle, meiosis, and longevity regulation pathways (Figure 7D).

Arthrobotrisins are structurally novel compounds produced in *A. oligospora*, and research has demonstrated that arthrobotrisins are associated with trap formation [46]. Therefore, the finding range was set to *m*/*z* = 393–394, and the WT strain detected the peak of arthrobotrisins at a retention time (Rt) of 30 min (diagnostic fragment ions at *m*/*z* 139, 393, and 429). However, no ionic peaks of arthrobotrisins were detected in the Δ*AosfgA* or Δ*AofluG* mutant strains (Figure S4A,B). The results suggest that the knockdown of *AosfgA* and *AofluG* genes affects the synthesis of arthrobotrisins.

## 4. Discussion

Asexual sporulation is an important aspect in the reproduction and environmental adaptation of filamentous fungi [22]. However, its complex regulatory mechanisms in NTFs are largely unknown. In the present research, we elucidated the functions and interactions of the key genes of the upstream regulatory network of conidium production, *AosfgA* and *AofluG*, in the model NTF *A. oligospora*.

SfgA is a Zn(II)2Cys6 family protein, a transcriptional regulator implicated in processes such as carbon and nitrogen utilization, secondary metabolism, and multicellular development [47]. The developmental activator *FluG* is necessary for asexual spore production as the core of UDAP [48]. Before asexual sporulation, *SfgA* proteins may repress the expression of *flbA*, *flbB*, and *flbC* by binding to promoters, whereas *FluG* proteins act as developmental activators participating in the synthesis of the extracellular sporulation-inducing factor [23,49]. According to previous studies, *sfgA* is located downstream of *fluG* [50]. In *A. nidulans*, double-mutant analyses of *sfgA* and *fluG* showed inhibition of conidia formation and sterigmatocystin production, and the overexpression of *sfgA* or deletion of *fluG* resulted in no conidium production [22]. In *A. oligospora*, we also verified the existence of an interaction between *AosfgA* and *AofluG* proteins.

In *A. nidulans* and *A. flavus*, *fluG* is the most upstream manager of growth and development [51]. The absence of *AosfgA* and *AosfgA* had a minimal effect on mycelial growth in *A. oligospora*, and there was no non-conidia-producing phenotype in the colonies of Δ*fluG* mutant strains, which is very different from *A. nidulans* [52]. Notably, we observed a considerable rise in the number of nuclei in hyphae but a decrease in the number of nuclei in spores in the Δ*AosfgA* and Δ*AofluG* mutant strains. Although mycelial growth, spore production, and pathogenicity defects were rare, the conidia of the Δ*AosfgA* and Δ*aofluG* mutants were altered in size, morphology, and complexity, which may also affect conidial function.

As mentioned previously, *fluG* is required to mediate spore production by generating extracellular secretion signals [20]. Meanwhile, Δ*fluG* mutant strains do not form conidiophores but produce spores under nutrient-deficient conditions [52]. In contrast, the Δ*sfgA* mutant strains produced more compact conidiophores [51]. In our study, spore production did not change significantly, but the conidiophores became more dense in the Δ*AosfgA* and Δ*AofluG* mutant strains. In addition, the proportion of immature conidia in the Δ*AosfgA* and Δ*AofluG* mutant strains was substantially greater than in the WT strains. Further, we detected a reduced level of *AofluG* gene expression in the Δ*AosfgA* mutant strain and an increased level of *AosfgA* expression in the Δ*AofluG* strain. Unlike *A. nidulans* and *A. flavus*, *AosfgA* may have some positive feedback on *AofluG* in *A. oligospora*, but overexpression and double mutation assays are needed to validate this and to draw definitive conclusions.

Fungal vacuoles have multiple roles in regulating cell growth and development, including ion metabolism and storage, pH and osmotic pressure regulation, nutrient transport, and apoptosis [53,54]. The process of autophagy, which transports intracellular cargo needing degradation to the vacuole, is closely linked to fungal development and pathogenicity [55]. In our results, an increase in vacuole volume was observed in both the Δ*AosfgA* and Δ*AofluG* mutant strains after nematode induction. Similarly, in our previous work, an increased vacuole volume, irregular shape, and elongation were observed after the knockdown of *AobrlA*, *AoabaA*, and *AowetA* genes in CDP [31]. However, our recent study also revealed that deletion of the *Aosec22* gene, which is associated with vesicle transport, results in the vacuoles in mycelial cells becoming small and fragmented in *A. oligospora* [42]. Notably, in *Drechslerella dactyloides*, the vacuoles in hyphae and uninflated ring cells of the Δ*DdVam7* mutant became smaller, also verifying that vacuole assembly is closely related to mycelial growth, conidiation, and the predatory process [56]. In summary, the vacuoles are critical for nutrient transport in fungi, and abnormal vacuole changes may interfere with trap maturation, while the formation of large vacuoles and the molecular mechanisms involved remain to be further explored.

The opposite expression levels of *AofluG* and *AosfgA* were verified via the RT-qPCR analysis of nine asexual spore-producing upstream regulatory genes in *A. oligospora*. Interestingly, the Δ*AosfgA* and Δ*AofluG* mutant strains also had different phenotypes to heat stress. Our results reveal that Δ*AosfgA* mutant strains are sensitive to high temperatures, while Δ*AofluG* mutant strains show insensitivity. Furthermore, we also verified that the Δ*AosfgA* and Δ*AofluG* mutant strains showed diverse phenotypes after H_2_O_2_, NaCl, sorbitol, and congo red treatments. Therefore, we inferred that the Δ*AosfgA* mutant strain could respond quickly upon sensing external stimuli compared to the Δ*AofluG* mutant strain. Similarly, in *A. flavus*, *sfgA* has been reported to have increased susceptibility to hyperosmotic, oxidative, and stressful pressures that are resistant to cell wall synthesis [51].

In *A. flavus*, deletion of the *sfgA* gene leads to increased aflatoxin biosynthesis, suggesting an essential role for *sfgA* in the regulation of secondary metabolism [51]. Using LC-MS analysis of the fermentation broth of the WT, Δ*AosfgA*, and Δ*AofluG* mutant strains, it was found that the abundance of compounds in the mutant strains was significantly decreased, and no arthrobotrisins peaks were detected. It is suggested that *AosfgA* and *AofluG* regulate secondary metabolism and are essential for arthrobotrisin biosynthesis. Meanwhile, KEGG enrichment analysis of the differential compounds revealed that *AosfgA* and *AofluG* are involved in multiple metabolic pathways. Based on metabolomics data, our results suggest that the *AosfgA* and *AofluG* genes can lead to a decrease in compound abundance in *A. oligospora* and affect the production of arthrobotrisins. Arthrobotrisins are one of the factors which impair trap development [46,57]. Meanwhile, G proteins [28] and the MAPK signaling pathway [30] play crucial roles in trap formation. In addition, trap formation is a very complex process regulated by multiple signaling pathways and cellular processes. Therefore, the effect of arthrobotrisins on traps may be counteracted by other cellular processes in this study. Collectively, our study proposes a novel modulation of *AosfgA* and *AofluG* in *A. oligospora* and reveals critical roles in mycelial and conidial development, stress responses, vacuole assembly, mitochondrial morphology, and secondary metabolism.

## Data Availability

All data generated or analyzed during this study are included in the published paper and associated Supplementary Materials.

## Supplementary Materials

The following supporting information can be downloaded at: https://www.mdpi.com/article/10.3390/microorganisms12030615/s1, Figure S1: Comparison of stress response to oxidative reagents between WT, Δ*AosfgA*, and Δ*AofluG* mutant strains; Figure S2: Comparison of stress response to osmotic reagents between WT, Δ*AosfgA*, and Δ*AofluG* mutant strains; Figure S3: Comparison of stress response to cell wall synthesis-disturbing reagents between WT, Δ*AosfgA* and Δ*AofluG* mutant strains; Figure S4: Detection of arthrobotrisins of WT, Δ*AosfgA*, and Δ*AofluG* mutant strains; Table S1: List of primers for RT-qPCR in this study; Table S2: List of primers for gene disruption in this study; Table S3: The top twenty compounds with significant changes in Δ*AosfgA* mutant strain; Table S4: The top twenty compounds with significant changes in Δ*AofluG* mutant strain.

## References

[1] Flores Francisco B.G., Ponce I.M., Plascencia Espinosa M.Á., Mendieta Moctezuma A., López Y López V.E. (2021). Advances in the biological control of phytoparasitic nematodes via the use of nematophagous fungi. World J. Microbiol. Biotechnol..

[2] Lahm G.P., Desaeger J., Smith B.K., Pahutski T.F., Rivera M.A., Meloro T., Kucharczyk R., Lett R.M., Daly A., Smith B.T. (2017). The discovery of fluazaindolizine: A new product for the control of plant parasitic nematodes. Bioorg. Med. Chem. Lett..

[3] Jiang X., Xiang M., Liu X. (2017). Nematode-trapping fungi. Microbiol. Spectr..

[4] Liu Q., Jiang K., Duan S., Zhao N., Shen Y., Zhu L., Zhang K.Q., Yang J. (2024). Identification of a transcription factor AoMsn2 of the Hog1 signaling pathway contributes to fungal growth, development and pathogenicity in *Arthrobotrys oligospora*. J. Adv. Res..

[5] Yang E., Xu L., Yang Y., Zhang X., Xiang M., Wang C., An Z., Liu X. (2012). Origin and evolution of carnivorism in the Ascomycota (fungi). Proc. Natl. Acad. Sci. USA.

[6] Luo H., Li X., Li G., Pan Y., Zhang K. (2006). Acanthocytes of *Stropharia rugosoannulata* function as a nematode-attacking device. Appl. Environ. Microbiol..

[7] Luo H., Liu Y., Fang L., Li X., Tang N., Zhang K. (2007). Coprinus comatus damages nematode cuticles mechanically with spiny balls and produces potent toxins to immobilize nematodes. Appl. Environ. Microbiol..

[8] Yang J., Liang L., Li J., Zhang K.Q. (2013). Nematicidal enzymes from microorganisms and their applications. Appl. Microbiol. Biotechnol..

[9] Liang L.M., Zou C.G., Xu J., Zhang K.Q. (2019). Signal pathways involved in microbe-nematode interactions provide new insights into the biocontrol of plant-parasitic nematodes. Philos. Trans. R. Soc. Lond. B Biol. Sci..

[10] Yang J., Wang L., Ji X., Feng Y., Li X., Zou C., Xu J., Ren Y., Mi Q., Wu J. (2011). Genomic and proteomic analyses of the fungus *Arthrobotrys oligospora* provide insights into nematode-trap formation. PLoS Pathog..

[11] Hsueh Y.P., Gronquist M.R., Schwarz E.M., Nath R.D., Lee C.H., Gharib S., Schroeder F.C., Sternberg P.W. (2017). Nematophagous fungus *Arthrobotrys oligospora* mimics olfactory cues of sex and food to lure its nematode prey. eLife.

[12] Singh R.K., Kumar N., Singh K.P. (2005). Morphological variations in conidia of *Arthrobotrys oligospora* on different media. Mycobiology.

[13] Dackman C., Nordbring-Hertz B. (1992). Conidial traps—A new survival structure of the nematode-trapping fungus *Arthrobotrys oligospora*. Mycol. Res..

[14] Persmark L., Nordbring-Hertz B. (1997). Conidial trap formation of nematode-trapping fungi in soil and soil extracts. FEMS Microbiol. Ecol..

[15] Diallo M., Kengen S.W.M., Lopez-Contreras A.M. (2021). Sporulation in solventogenic and acetogenic clostridia. Appl. Microbiol. Biotechnol..

[16] Jung B., Kim S., Lee J. (2014). Microcyle conidiation in filamentous fungi. Mycobiology.

[17] Boylan M.T., Mirabito P.M., Willett C.E., Zimmerman C.R., Timberlake W.E. (1987). Isolation and physical characterization of three essential conidiation genes from *Aspergillus nidulans*. Mol. Cell Biol..

[18] Clutterbuck A.J. (1969). A mutational analysis of conidial development in *Aspergillus nidulans*. Genetics.

[19] Adams T.H., Boylan M.T., Timberlake W.E. (1988). brlA is necessary and sufficient to direct conidiophore development in *Aspergillus nidulans*. Cell.

[20] Lee B.N., Adams T.H. (1994). The *Aspergillus nidulans fluG* gene is required for production of an extracellular developmental signal and is related to prokaryotic glutamine synthetase I. Genes Dev..

[21] Wieser J., Lee B.N., Fondon J., Adams T.H. (1994). Genetic requirements for initiating asexual development in *Aspergillus nidulans*. Curr. Genet..

[22] Seo J.A., Guan Y., Yu J.H. (2006). FluG-dependent asexual development in *Aspergillus nidulans* occurs via derepression. Genetics.

[23] Park H.S., Yu J.H. (2012). Genetic control of asexual sporulation in filamentous fungi. Curr. Opin. Microbiol..

[24] Krijgsheld P., Nitsche B.M., Post H., Levin A.M., Muller W.H., Heck A.J., Ram A.F., Altelaar A.F., Wosten H.A. (2013). Deletion of *flbA* results in increased secretome complexity and reduced secretion heterogeneity in colonies of *Aspergillus niger*. J. Proteome Res..

[25] Krijgsheld P., Bleichrodt R., van Veluw G.J., Wang F., Muller W.H., Dijksterhuis J., Wosten H.A. (2013). Development in *Aspergillus*. Stud. Mycol..

[26] Ruger-Herreros C., Rodriguez-Romero J., Fernandez-Barranco R., Olmedo M., Fischer R., Corrochano L.M., Canovas D. (2011). Regulation of conidiation by light in *Aspergillus nidulans*. Genetics.

[27] D’Souza C.A., Lee B.N., Adams T.H. (2001). Characterization of the role of the FluG protein in asexual development of *Aspergillus nidulans*. Genetics.

[28] Yang X., Ma N., Yang L., Zheng Y., Zhen Z., Li Q., Xie M., Li J., Zhang K.Q., Yang J. (2018). Two Rab GTPases play different roles in conidiation, trap formation, stress resistance, and virulence in the nematode-trapping fungus *Arthrobotrys oligospora*. Appl. Microbiol. Biotechnol..

[29] Zhu Y., Yang X., Bai N., Liu Q., Yang J. (2024). AoRab7A interacts with AoVps35 and AoVps41 to regulate vacuole assembly, trap formation, conidiation, and functions of proteasomes and ribosomes in *Arthrobotrys oligospora*. Microbiol. Res..

[30] Xie M., Bai N., Yang X., Liu Y., Zhang K.Q., Yang J. (2023). Fus3 regulates asexual development and trap morphogenesis in the nematode-trapping fungus *Arthrobotrys oligospora*. iScience.

[31] Bai N., Xie M., Liu Q., Zhu Y., Yang X., Zhang K.Q., Yang J. (2023). AoMedA has a complex regulatory relationship with AoBrlA, AoAbaA, and AoWetA in conidiation, trap formation, and secondary metabolism in the nematode-trapping fungus *Arthrobotrys oligospora*. Appl. Environ. Microbiol..

[32] Liu Q., Li D., Bai N., Zhu Y., Yang J. (2023). Peroxin Pex14/17 is required for trap Formation, and plays pleiotropic roles in mycelial development, stress response, and secondary metabolism in *Arthrobotrys oligospora*. mSphere.

[33] Cui P., Tian M., Huang J., Zheng X., Guo Y., Li G., Wang X. (2022). Amphiphysin AoRvs167-mediated membrane curvature facilitates trap formation, endocytosis, and stress resistance in *Arthrobotrys oligospora*. Pathogens.

[34] Livak K.J., Schmittgen T.D. (2001). Analysis of relative gene expression data using real-time quantitative PCR and the 2(-Delta Delta C(T)) Method. Methods.

[35] Zientara-Rytter K., Ozeki K., Nazarko T.Y., Subramani S. (2018). Pex3 and Atg37 compete to regulate the interaction between the pexophagy receptor, Atg30, and the Hrr25 kinase. Autophagy.

[36] Chen S.A., Lin H.C., Hsueh Y.P. (2022). The cAMP-PKA pathway regulates prey sensing and trap morphogenesis in the nematode-trapping fungus *Arthrobotrys oligospora*. G3.

[37] Kumar S., Stecher G., Tamura K. (2016). MEGA7: Molecular evolutionary genetics analysis version 7.0 for bigger datasets. Mol. Biol. Evol..

[38] Xu J., Li J., Lin L., Liu Q., Sun W., Huang B., Tian C. (2015). Development of genetic tools for *Myceliophthora thermophila*. BMC Biotechnol..

[39] Zhao X., Fan Y., Xiang M., Kang S., Wang S., Liu X. (2022). DdaCrz1, a C(2)H(2)-type transcription factor, regulates growth, conidiation, and stress resistance in the nematode-trapping fungus *Drechslerella dactyloides*. J. Fungi.

[40] Xiao J., Zhang Y., Yang K., Tang Y., Wei L., Liu E., Liang Z. (2022). Protein kinase Ime2 is associated with mycelial growth, conidiation, osmoregulation, and pathogenicity in *Fusarium oxysporum*. Arch. Microbiol..

[41] Liu X., Miao Q., Zhou Z., Lu S., Li J. (2022). Identification of three novel conidiogenesis-related genes in the nematode-trapping fungus *Arthrobotrys oligospora*. Pathogens.

[42] Zhou L., Li M., Cui P., Tian M., Xu Y., Zheng X., Zhang K., Li G., Wang X. (2022). Arrestin-coding genes regulate endocytosis, sporulation, pathogenicity, and stress resistance in *Arthrobotrys oligospora*. Front. Cell. Infect. Microbiol..

[43] Gu T., Lu H., Liu H., Zhang G., Wang Y. (2023). Function discovery of a non-ribosomal peptide synthetase-like encoding gene in the nematode-trapping fungus *Arthrobotrys oligospora*. Front. Microbiol..

[44] Zhang Y., Wang X., Ran Y., Zhang K.Q., Li G.H. (2023). AfLaeA, a global regulator of mycelial growth, chlamydospore production, pathogenicity, secondary metabolism, and energy metabolism in the nematode-trapping fungus *Arthrobotrys flagrans*. Microbiol. Spectr..

[45] Yang X., Chen Y., Zhang L., He J., Wu Q., Li S., Wang D., Gou J., Wu Z., Zhang K. (2023). Melanin precursors mediated adaption to temperature changes in fungus and animal via inhibition of lipid-mediated ferroptosis. Sci. China Life Sci..

[46] He Z.Q., Tan J.L., Li N., Zhang H.X., Chen Y.H., Wang L.J., Zhang K.Q., Niu X.M. (2019). Sesquiterpenyl epoxy-cyclohexenoids and their signaling functions in nematode-trapping fungus *Arthrobotrys oligospora*. J. Agric. Food Chem..

[47] Todd R.B., Andrianopoulos A. (1997). Evolution of a fungal regulatory gene family: The Zn(II)2Cys6 binuclear cluster DNA binding motif. Fungal Genet. Biol..

[48] Ojeda-Lopez M., Chen W., Eagle C.E., Gutierrez G., Jia W.L., Swilaiman S.S., Huang Z., Park H.S., Yu J.H., Canovas D. (2018). Evolution of asexual and sexual reproduction in the *aspergilli*. Stud. Mycol..

[49] Lee M.K., Kwon N.J., Lee I.S., Jung S., Kim S.C., Yu J.H. (2016). Negative regulation and developmental competence in *Aspergillus*. Sci. Rep..

[50] Rodriguez-Urra A.B., Jimenez C., Nieto M.I., Rodriguez J., Hayashi H., Ugalde U. (2012). Signaling the induction of sporulation involves the interaction of two secondary metabolites in *Aspergillus nidulans*. ACS Chem. Biol..

[51] Yuan X.Y., Li J.Y., Zhi Q.Q., Chi S.D., Qu S., Luo Y.F., He Z.M. (2022). SfgA renders *Aspergillus flavus* more stable to the external environment. J. Fungi.

[52] Adams T.H., Hide W.A., Yager L.N., Lee B.N. (1992). Isolation of a gene required for programmed initiation of development by *Aspergillus nidulans*. Mol. Cell Biol..

[53] Chavrier P., Parton R.G., Hauri H.P., Simons K., Zerial M. (1990). Localization of low molecular weight GTP binding proteins to exocytic and endocytic compartments. Cell.

[54] Klionsky D.J., Herman P.K., Emr S.D. (1990). The fungal vacuole: Composition, function, and biogenesis. Microbiol. Rev..

[55] Gao H.M., Liu X.G., Shi H.B., Lu J.P., Yang J., Lin F.C., Liu X.H. (2013). MoMon1 is required for vacuolar assembly, conidiogenesis and pathogenicity in the rice blast fungus *Magnaporthe oryzae*. Res. Microbiol..

[56] Chen Y., Liu J., Fan Y., Xiang M., Kang S., Wei D., Liu X. (2022). SNARE protein DdVam7 of the nematode-trapping fungus *Drechslerella dactyloides* regulates vegetative growth, conidiation, and the predatory process via vacuole assembly. Microbiol. Spectr..

[57] Yu X., Hu X., Pop M., Wernet N., Kirschhöfer F., Brenner-Weiß G., Keller J., Bunzel M., Fischer R. (2021). Fatal attraction of *Caenorhabditis elegans* to predatory fungi through 6-methyl-salicylic acid. Nat. Commun..

