# Supplementary material for: Characterizing the Role of AosfgA and AofluG in Mycelial and Conidial Development in Arthrobotrys oligospora and Their Role in Secondary Metabolism
Source: Microorganisms. 2024 Mar 19;12(3):615. doi: 10.3390/microorganisms12030615 (PMC10975216; doi:10.3390/microorganisms12030615)
Supplement: Supplementary file 1 [file microorganisms-12-00615-s001.zip › microorganisms-2882245-supplementary.pdf]

Supporting Information

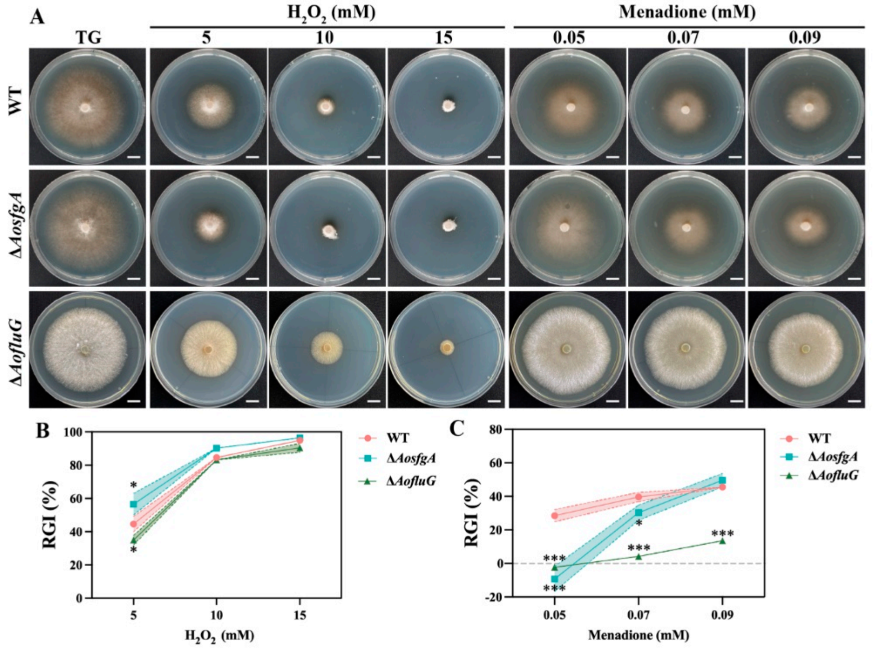

Figure S1. Comparison of stress response to oxidative reagents between WT, Δ*AosfgA* and Δ*AofluG* mutant strains. **(A)** Colony morphology on the medium supplemented with different oxidants. Bar = 1 cm. **(B, C)** Comparison of RGI values under H_2O_2 (B) and menadione (C). Asterisks indicate that the mutant strain significantly differs from the WT strain (Tukey's HSD, **p* < 0.05, ****p* < 0.001).

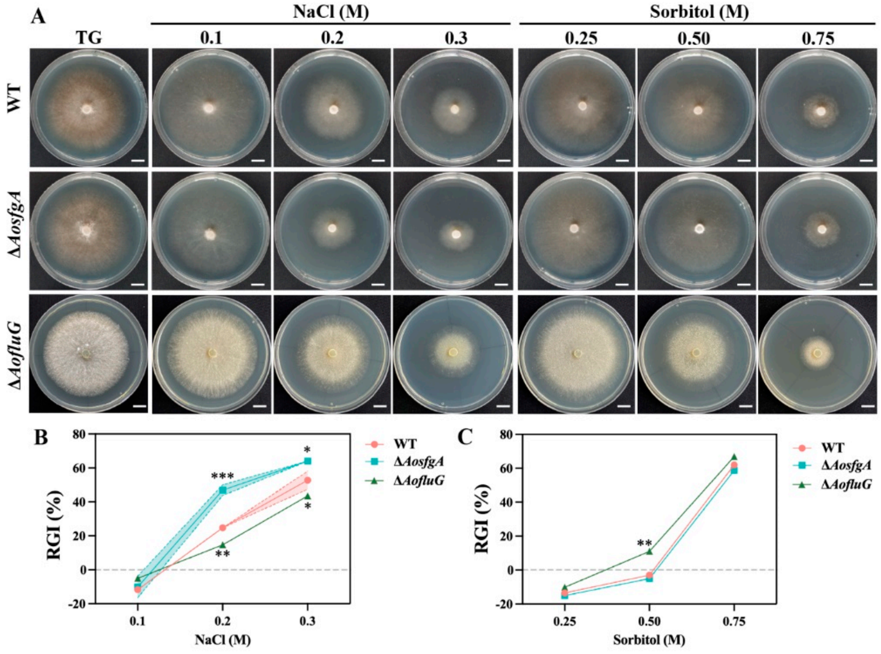

Figure S2. Comparison of stress response to osmotic reagents between WT, Δ*AosfgA* and Δ*AofluG* mutant strains. **(A)** Colony morphology on medium supplemented with different high osmotic chemical reagents. Bar = 1 cm. **(B, C)** Comparison of RGI values under NaCl (B) and sorbitol (C). Asterisks indicate that the mutant strain significantly differs from the WT strain (Tukey's HSD, **p* < 0.05, ***p* < 0.01, ****p* < 0.001).

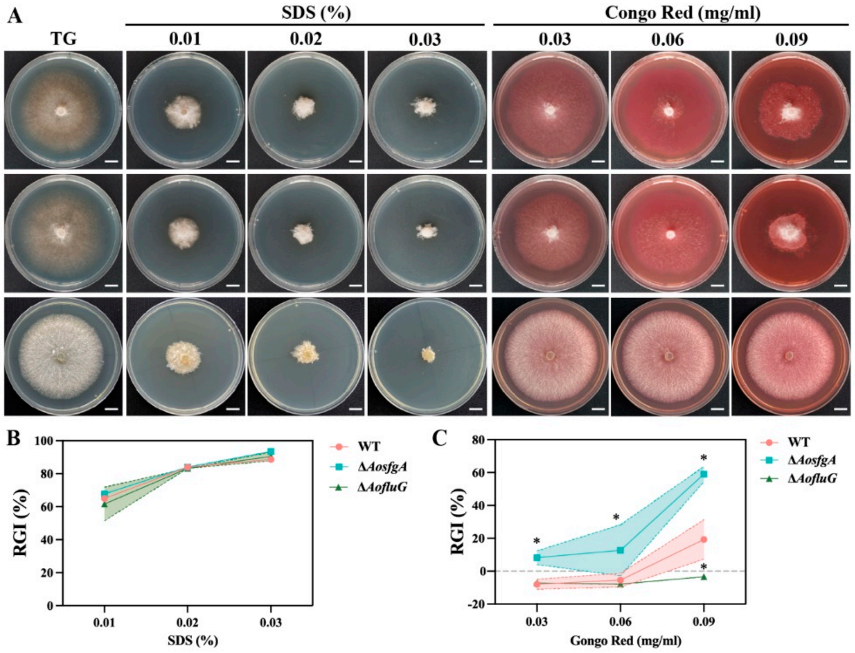

Figure S3. Comparison of stress response to cell wall synthesis-disturbing reagents between WT, Δ*AosfgA* and Δ*AofluG* mutant strains. **(A)** Colony morphology on medium supplemented with different cell wall synthesis-disturbing reagents. Bar = 1 cm. **(B, C)** Comparison of RGI values under SDS (B) and congo red (C). Asterisks indicate that the mutant strain significantly differs from the WT strain (Tukey's HSD, *$p < 0.05$).

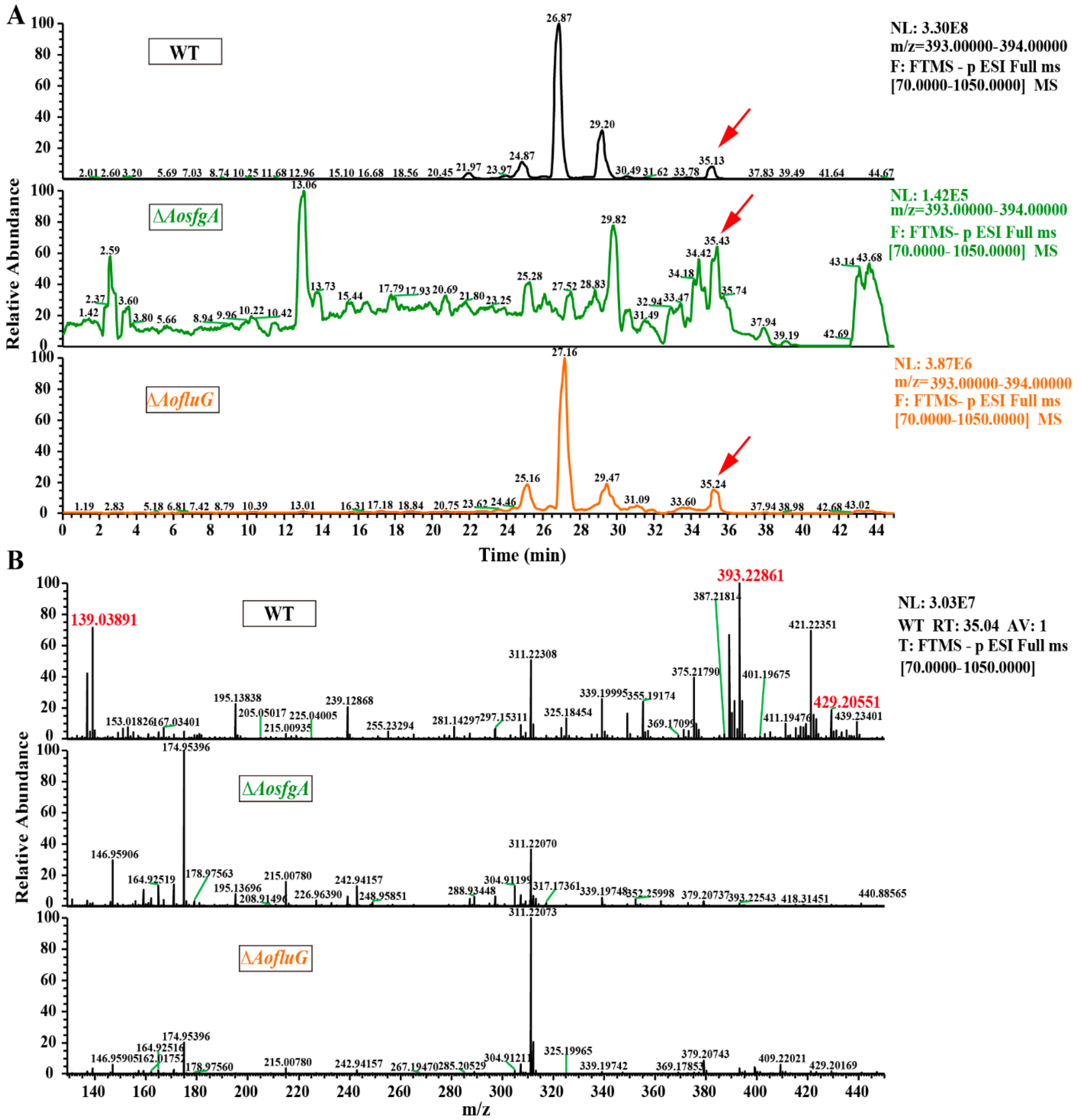

Figure S4. Detection of arthrobotrisins of WT, Δ*AosfgA* and Δ*AofluG* mutant strains. **(A)** Chromatographic analysis of arthrobotrisins in WT, Δ*AosfgA* and Δ*AofluG* mutant strains. **(B)** Mass spectrum analysis of arthrobotrisins in WT, Δ*AosfgA* and Δ*AofluG* mutant strains. The diagnostic fragment ions (at m/z 139, 393, and 429) of arthrobotrisins was found in the WT strain.

Table S1. List of primers for RT-qPCR in this study.

Primers	Sequence (5′-3′)	Sequence (3′-5′)
**Sporulation-related genes**		
AOL_s00043g361 (*AofluG*)	GATTCCAGTCCCGTGAATTC	GCTAAGGAGAGGATGGGCAT
AOL_s00097g406 (*AosfgA*)	CTCCAACAATGAGGCCGAGA	GGGCTGCGGACAAGGATAAA
AOL_s00215g516 (*AoflbA*)	TTCAAACGCAGCTCCTTCAC	AAGCGGGTTGACAGATGAGA

AOL_s00078g317 (*AoflbB*)	AGCATATCCAGAAACTTGTCCA	TAGAACCCCAATCAATATCCGG
AOL_s00007g157 (*AoflbC*)	CTCTCCGGCAAAGACAATCG	GTCGACTGAGGATAGTAGCT
AOL_s00097g46 (*AoflbD*)	TAAAAGGCAGAAGTGACAATGC	CGATGGGATAAGAGTTGGATGA
AOL_s00215g48 (*AofphA*)	AATATCCTGCCATACTTACGGG	CTCTCAATATTTCGACTTGCGG
AOL_s00054g700 (*AovosA*)	CAAACCACCCACCACCAAAT	GGATGGACAGGAGAAGGACC
AOL_s00054g811 (*AovelB*)	ATTCCGCAACTTCTCCCTCA	GGCATGTTTGGATTCTGGGG
AOL_s00169g18 (*AoveA*)	AAGCTACACCCAATCAACGC	TTGCGATGCTGACGATCTTG
AOL_s00075g211 (*AonsdD*)	ATTACGGCCGCCTAGTAGTC	CTCGTTTGGACCTGGTTGTG
AOL_s00006g570 (*Aohyp1*)	GCGGATCCAACATGAAGCTT	GGTTGACAACTGGGATGCTG
AOL_s00080g63 (*AoabaA*)	AACTTTATGCGCCTTGTCGT	TTGGCTAGGTGGTCTGTACG
AOL_s00079g294 (*Aoste12*)	GCTACACCTCGCTATGCCAA	ATTGTTTCAGTCGCAGCACG
AOL_s00083g25 (*AostuA*)	AGCTCCCGAAACGAGTCTAA	ATTGATCATGTGATTATCCT
AOL_s00097g514 (*AobrlA*)	TTGAGGCCTCGATCCGTAGA	AGGTAGATGGCGCTGTTACG
AOL_s00173g221 (*AowetA*)	TTACATGCCACCCCAAGTCC	CAATTGCAACTGCGTCCACA
AOL_s00210g120 (*AomedA*)	TCCGGCCCAATGATTCAGAA	AGATCGCAGGAACATGGTGA
AOL_s00080g93 (*AolreB*)	CCAGGGTCGTCAGTATCTT	CAGCATCTTCCAGGTCAA
**Internal control**		
AOL_s00076g640 (*tub*)	CCACCTTCGTCGGTAACTC	TCGTCCATACCCTCACCAG

Table S2. List of primers for gene disruption in this study.

Primers	Sequence (5′-3′)
**Amplify the *AoSfgA* gene 5′ flank**	
AoSfgA-FF	GTAACGCCAGGGTTTTCCCAGTCACGACGGCGCAAATTTGTCAGTTGGC
AoSfgA-FR	ATCCACTTAACGTTACTGAAATCTCCAACGAGCAGGGACGAAAACTCTCA
**Amplify the *AoSfgA* gene 3′ flank**	
AoSfgA-RF	CTCCTTCAATATCATCTTCTGTCTCCGACAATGAACTCGCTGAACTCGC
AoSfgA-RR	GCGGATAACAATTTCACACAGGAAACAGCGAAAACCCGCCCTCTCGAA
**Amplify the *AofluG* gene 5′ flank**	
AofluG-FF	GTAACGCCAGGGTTTTCCCAGTCACGACGGTTAAGCGCTCTCATATTCTCC
AofluG -FR	ATCCACTTAACGTTACTGAAATCTCCAACAGATCCCGAACTATTTACCAGG
**Amplify the *AofluG* gene 3′ flank**	
AofluG -FF	CTCCTTCAATATCATCTTCTGTCTCCGACCGAACCAGCTATGAGGAACG
AofluG -FR	GCGGATAACAATTTCACACAGGAAACAGCTACCCGTAAACTGATTCATCG
**Amplify the *hph* cassette**	
Hph-F	GTCGGAGACAGAAGATGATATTGAAGGAGC
Hph-R	GTTGGAGATTTCAGTAACGTTAAGTGGAT
**Verify the transformants**	
AoSfgA-YZ-F	TGAGAGTTTTCGTCCCTGCTC
AoSfgA-YZ-R	GCGAGTTCAGCGAGTTCATT
AofluG-YZ-F	GTTAAGCGCTCTCATATTCTCC
AofluG-YZ-R	TACCCGTAAACTGATTCATCG
**Make Southern blotting probe**	
AoSfgA-TZ-F	CCCCCAAAGAGCAACCGTAA
AoSfgA-TZ-R	GAGCAGGGACGAAAACTCTCA
AofluG-TZ-F	CTGCACCCGTTTCGATTTCG

AofluG-TZ-R	GGTCTTCCTTTTCTTTAATC

Table S3. The top twenty compounds with significant changes in Δ*AosfgA* mutant strain.

Name	Formula	Molecular Weight	RT (min)	Log2 Fold Change: Δ*AosfgA*/WT	P-value: Δ*AosfgA*/WT
Prednisone	$C_{21}H_{26}O_5$	358.17724	35.799	-9.74	0.049952
Diamino-N-carbamoylmethaniminium	$C_2H_7N_4O$	103.06122	2.375	4.56	0.04994505
2'-(Ethylsulfanyl)-7',7'-dimethyl-5'-oxo-5',6',7',8'-tetrahydro-1'H-spiro[cyclohexane-1,4'-quinoline]-3'-carbonitrile	$C_{19}H_{26}N_2OS$	330.17787	34.543	2.11	0.04976551
2-hydroxy palmitic acid	$C_{16}H_{32}O_3$	272.23472	41.169	-4.07	0.04974411
[FA (18:4)]6Z_9Z_12Z_15Z-octadecatetraenoicacid	$C_{18}H_{28}O_2$	276.20826	37.006	-5.26	0.04956678
9-Keto heptadecylic acid	$C_{17}H_{32}O_3$	284.23454	41.105	-7.22	0.04954504
3,4-Methylenedioxy-2',4'-dimethoxychalcone	$C_{18}H_{16}O_5$	312.09903	34.003	-6.16	0.04954233
UNII:212JQJ15PS	C_8H_{12}	108.09399	37.469	-3.21	0.04953087
N-arachidonoylethanolamine	$C_{22}H_3NO_2$	347.28166	41.441	-4.72	0.04944926
Cyclo(glycylprolylglycylprolylglycylprolyl)	$C_{21}H_{30}N_6O_6$	462.22426	33.176	-3.41	0.04940738
Malyngic acid	$C_{18}H_{32}O_5$	328.22431	33.425	-3.84	0.04925605
3-[(4-Chlorophenyl) sulfanyl]-6-methoxy-N-[3-(4-methyl-1-piperazinyl) propyl]-1H-indole-2-carboxamide	$C_{24}H_{29}ClN_4O_2S$	472.16765	29.547	-5.69	0.04919087
Euchrenone a14	$C_{30}H_{32}O_5$	472.22394	42.87	-3.67	0.04917107
2,6-DIMETHYLNAPHTHALENE	$C_{12}H_{12}$	156.09361	36.165	-4.59	0.0491285
Î±-Calacorene	$C_{15}H_{20}$	200.1561	31.246	-6.42	0.04907292
Panaxynol	$C_{17}H_{24}O$	244.18227	38.672	-7.64	0.04892482
4S-hydroperoxy-17S-HDHA	$C_{22}H_{32}O_5$	376.2241	40.336	-9.83	0.04888879
6,3',4'-Trihydroxy-4-methoxy-5-methylaurone	$C_{17}H_{14}O_6$	314.07835	33.917	-7.12	0.0488716
3,6,9,12,15-octadecapentaenoic acid	$C_{18}H_{26}O_2$	274.19271	40.415	-5.74	0.04885755
Auricolic acid	$C_{20}H_{36}O_3$	324.26583	40.792	-2.37	0.04885534

Table S4. The top twenty compounds with significant changes in Δ*AofluG* mutant strain.

Name	Formula	Molecular Weight	RT (min)	Log2 Fold Change: Δ*AofluG*/WT	P-value: Δ*AofluG*/WT
Trans-Non-2-en-(4.6.8)-triyn-1-ol	C_9H_6O	130.04186	10.164	-3.84	0.04999131
3'-Angeloyloxy-2',4'-dihydroxy-6'-methoxychalcone	$C_{21}H_{20}O_6$	368.12516	32.788	-4.81	0.04981307
Trichurusin F	$C_{25}H_{36}O_7$	448.24465	36.963	-6.84	0.04979714

12,13-dihydroxy-11-methoxy-9-octadecenoic acid	$C_{19}H_{36}O_5$	344.25549	35.012	-8.84	0.0497816
DQ6125000	$C_{14}H_{14}O$	198.10416	31.429	-2.09	0.04975198
1H-Imidazole-4,5-dicarbohydrazide	$C_5H_8N_6O_2$	184.07044	8.797	-4.16	0.04972341
CZ1330000	$C_{12}H_{16}$	160.12498	10.392	-2.2	0.04963597
Callyspongenol C	$C_{22}H_{24}O$	304.18187	33.137	-5.72	0.04961497
Berkazaphilone A	$C_{13}H_{16}O_3$	220.10954	40.575	-5.56	0.04958738
Grandiflorone	$C_{19}H_{22}O_4$	314.15126	33.426	-2.64	0.04954353
12S-hydroxy-16-heptadecynoic acid	$C_{17}H_{30}O_3$	282.21888	39.862	-8.47	0.04953914
naphthalen-1-ethanol	$C_{12}H_{12}O$	172.08855	17.434	-2.38	0.04952013
7,8,9,10-tetrahydro-9-hydroxy-4-methoxy-9-propyltetracene-6,11-dione	$C_{22}H_{22}O_4$	350.15101	32.857	-5.93	0.04949931
Deoxycorticosterone Acetate	$C_{23}H_{32}O_4$	372.2289	40.875	-8.03	0.04949851
2,2-Diphenylcyclopentanone	$C_{17}H_{16}O$	236.11963	18.766	-4.41	0.04936962
Brosimacutin G	$C_{20}H_{20}O_6$	356.12553	24.165	-2.83	0.04920574
Dihydrochalcone	$C_{15}H_{14}O$	210.10388	31.847	-2.31	0.04917659
4'-O-Methylbavachalcone	$C_{22}H_{24}O_4$	352.1668	24.108	-2.41	0.04911524
Benzoin	$C_{14}H_{12}O_2$	212.08326	15.401	-2.55	0.04907194
35-O-β– 3,5-anhydrogalacturonopyranosylbacteriohopanetetrol	$C_{41}H_{68}O_9$	704.48475	38.647	-4.82	0.04906659
